# High Prevalence of Quorum-Sensing and Quorum-Quenching Activity among Cultivable Bacteria and Metagenomic Sequences in the Mediterranean Sea

**DOI:** 10.3390/genes9020100

**Published:** 2018-02-16

**Authors:** Andrea Muras, Mario López-Pérez, Celia Mayer, Ana Parga, Jaime Amaro-Blanco, Ana Otero

**Affiliations:** 1 Departamento de Microbioloxía e Parasitoloxía, Facultade de Bioloxía-CIBUS, Universidade de Santiago de Compostela, Santiago de Compostela 15782, Spain; andrea.muras@usc.es (A.M.); celiammayer@gmail.com (C.M.); anapargamartinez@yahoo.es (A.P.); jaimeamaro85@gmail.com (J.A.-B.); 2 Evolutionary Genomics Group, División de Microbiología, Universidad Miguel Hernández, San Juan de Alicante 03202, Spain; mario.lopezp@umh.es

**Keywords:** quorum sensing, quorum quenching, AHL, lactonase, acylase, marine bacteria

## Abstract

There is increasing evidence being accumulated regarding the importance of *N*-acyl homoserine lactones (AHL)-mediated quorum-sensing (QS) and quorum-quenching (QQ) processes in the marine environment, but in most cases, data has been obtained from specific microhabitats, and subsequently little is known regarding these activities in free-living marine bacteria. The QS and QQ activities among 605 bacterial isolates obtained at 90 and 2000 m depths in the Mediterranean Sea were analyzed. Additionally, putative QS and QQ sequences were searched in metagenomic data obtained at different depths (15–2000 m) at the same sampling site. The number of AHL producers was higher in the 90 m sample (37.66%) than in the 2000 m sample (4.01%). However, the presence of QQ enzymatic activity was 1.63-fold higher in the 2000 m sample. The analysis of putative QQ enzymes in the metagenomes supports the relevance of QQ processes in the deepest samples, found in cultivable bacteria. Despite the unavoidable biases in the cultivation methods and biosensor assays and the possible promiscuous activity of the QQ enzymes retrieved in the metagenomic analysis, the results indicate that AHL-related QS and QQ processes could be common activity in the marine environment.

## 1. Introduction

Quorum sensing (QS) is a bacterial communication system based on the production and secretion of small signal molecules called autoinducers that accumulate in the extracellular environment when high cell densities are reached [1]. Once a threshold intracellular concentration is achieved, the signaling molecule triggers the synchronous expression of multiple genes in the population, initiating a coordinated action. Although different types of signal molecules have been described [2], the best characterized QS signals are the *N*-acyl homoserine lactones (AHLs). These QS signal molecules are constituted by a homoserine lactone ring (HSL) linked by an amide bond to a fatty acid (between 4 and 18 carbons). AHLs are QS signals that are considered typical of Gram-negative bacteria [3], although they are also produced by different clades of bacteria [4,5], including the Gram-positive *Exiguobacterium* sp. [6]. The most common AHL-based QS system comprises a LuxI-type signal synthase and a LuxR-type receptor [7]. Other AHL synthases belonging to the families LuxM/AinS and HdtS have been described, not sharing homology with the LuxI synthase family [8,9]. Some bacteria do not produce AHLs or have a recognizable LuxI autoinducer synthase but possess LuxR homologs, called LuxR orphans, that can interact with the autoinducers synthetized by other bacteria [10]. Recently, LuxR homologues have been described to act as sensors for QS signals different from AHLs, making the picture more complex [11]. Despite being more infrequently reported, LuxI orphans are also present in some bacteria [12].

In spite of the low bacterial population in the open sea and the low chemical stability of AHLs at the high pH of seawater, new evidence reinforces the idea of the importance of AHL-mediated QS mechanisms in marine environments [3,5,13,14]. More recently, numerous studies have reported the isolation of AHL-producing bacterial strains from marine samples [15,16,17,18,19,20]. The presence of bacteria with the ability to produce AHLs in these marine microhabitats was reported in subtidal biofilms [21], sponges [16,22], cnidarians [23,24], and marine snow [15,19,25]. It is now generally accepted that the AHL-mediated QS systems play an important role in relevant marine ecology processes including the settlement of invertebrate larvae [26,27] and of macroalgae zoospores [28]. The AHLs produced by bacteria associated with the cyanobacteria *Trichodesmium* are proposed to mediate and coordinate the processing and acquisition of phosphorus [29], a limiting nutrient in oligotrophic open ocean environments. Furthermore, symbiotic and pathogenic interactions with a eukaryotic host also act as examples of these ecologically relevant niches [28,30]. In addition, the expression of important virulence genes in marine fish pathogenic bacteria is commonly controlled through AHL-mediated processes [31]. 

The presence of AHLs in open marine environments has been also reported using direct, non-cultivation-dependent measurements [25,32]. A large phylogenetic diversity of the AHLs synthases was observed in the Global Ocean Sampling (GOS) metagenomic database [33], suggesting that AHL production is a widespread mechanism in marine environments. The AHLs are proposed to participate in the marine carbon cycle by increasing the activity of certain key hydrolytic enzymes for the degradation of particulate organic carbon in seawater, playing an important role in the remineralisation depth distribution of sinking particulate organic carbon (POC) [25,34]. QS-mediated processes have been suggested to be even more ecologically relevant in specific marine microhabitats in which the bacterial population is more concentrated, forming cell clusters [13,14]. 

Since QS systems have important effects in the interactions between prokaryotes and also with eukaryotes, it makes sense that competitors have evolved mechanisms for silencing other bacterial QS systems. The ability to disrupt bacterial communication is a widespread strategy used by different kinds of organisms: marine algae [35], terrestrial plants [36], mammalian cells [37], and bacteria [4,38,39]. The term quorum quenching (QQ) was coined to describe the enzymatic inactivation of AHL QS signals [40], although at present this term is often used in a general sense to describe any type of QS disruption [41]. Enzymatic QQ is the best studied QS inhibitory strategy [40]. The genes that codify this type of enzymes are classified in two main groups: lactonases and acylases, although other types of QQ enzymes have also been described [39]. The pioneer studies on the ecological relevance of QQ processes carried out with bacteria isolated from soil and rhizosphere indicated that 2–4.8% of these strains had the ability to interfere with AHLs [42,43,44]. More recent studies revealed a high prevalence of QQ enzymes in the marine environment: enzymatic QQ activity was observed in bacterial strains isolated from corals [23,45], sponges [46], marine biofilms [47], estuarine and open ocean superficial seawater [48,49], and fish and bivalve hatcheries [50,51], presenting higher frequencies of bacteria with this capability (2–46%) in comparison to terrestrial samples [46,48,52,53,54]. The importance of QQ processes in the marine environment was further supported by metagenomic studies showing a high frequency of QQ enzymes in marine metagenomic collections including the Global Ocean Sampling collection [48]. Despite the relevance of QS and QQ in niche marine environments seems clear and all the available data points to a high prevalence of QS and QQ activities in the seawater, there is no study in which a metagenomic analysis is combined with the analysis of the QS and QQ activities among cultivable isolates for the same sample. This double approach would allow us to avoid the handicaps of both methods and to assess the relevance of these processes in free-living bacteria. Therefore, the aim of this work was to study the AHL production and degradation activity in free-living bacteria from the Mediterranean Sea using two different but complementary approaches, such as functional screening in bacteria able to grow in standard culture conditions and metagenomic analysis in order to improve our understanding of the ecologic relevance of AHL-mediated processes in the marine environment. The metagenomic analysis was carried out from seawater samples collected from six depths in the photic zone at 15 m intervals (15, 30, 45, 60, 75, and 90 m) and from two depths in the aphotic zone (1000 and 2000 m) at the same time, while cultivable bacteria were obtained from the 90 and 2000 m samples in order to assess the spatial distribution of the QS and QQ processes.

## 2. Materials and Methods 

### 2.1. Sample Collection, Bacterial Quantification, and Strain Isolation

Eight seawater samples from different depths were collected for metagenomic analyses as described previously [55,56] on October 15, 2015 at a single point in the Mediterranean Sea (37.35361° N, 0.286194° W) by the research vessel ‘García del Cid’. Samples from 90 and 2000 m depth were also used for bacterial isolation and functional screening. The 90 m sample is considered the limit of the photic zone, with a chlorophyll-a (Chl-a) value of 0.13 mg/m^3^, a total organic carbon (TOC) of 1.35 mgC/L, total N of 6.9 µM, total P of 0.25 µM, and 1.37 × 10^5^ heterotrophic bacteria counts. The 2000 m sample was characterized by an almost undetectable Chl-a concentration (0.01 mg/m^3^), lower TOC (0.94 mg C/L), higher total N and *p*-values (8.62 and 0.5 µM, respectively), and lower heterotrophic bacteria counts (4.5 × 10^4^) [55,56]. Both rich and oligotrophic culture media were used for bacterial isolation as previously described [47]. The rich media included tryptone soy agar 1% NaCl (TSA-1) and marine agar (MA) suitable for eutrophic bacteria, and the low organic formulations included MA diluted 1/100 with seawater (salinity 35 g/L) and filtered autoclaved seawater medium (FAS) supplemented with 0.5 g/L of each of the following polymers: agarose, chitin, and starch (FAS-POL). Five series of 10-fold dilutions were prepared in sterilized natural seawater for each sample and plated in the above-mentioned culture media. The plates were incubated at 22 °C for 15 days. For the estimation of colony forming units (CFUs), plates with 30–300 colonies were selected. A total of 605 strains were randomly picked up and isolated to be used for QS and QQ functional screening (Table S1). All the marine isolates obtained were able to grow on MA at 22 °C, hence these culture conditions were selected as standard for laboratory maintenance and assays.

### 2.2. 16S-Based Bacterial Identification

The identification of the collection of 605 cultivable marine bacteria was carried out using 200 μL of cultures obtained in marine broth and pooled in groups of 65–80 strains. These pools were centrifuged, and the DNA was extracted with DNeasy PowerSoil Kit (Qiagen^®^, Hilden, Germany). The amplification of 16S rRNA genes was performed using a diversity assay illumine inhouse bTEFAP^®^ (Lubbock, TX, USA). PCR were carried out under the following standard conditions: initial step of 94 °C for 3 min followed by 28 cycles at 94 °C for 30 s, 53 °C for 40 s, and 72 °C for 1 min. Sequencing (Miseq, Illumina, San Diego, CA, USA) and data processing were performed using BLASTn against a curated database from RDPII and NCBI (MR DNA, Shallowater, TX, USA).

In order to analyze the bacterial diversity of the cultivable bacteria, the 16S rDNA sequences were clustered to operational taxonomic units (OTUs) defined at 95% identity using CD-HIT [57]. The sequences were assigned a taxonomic identity using the RDP database [58].

For the isolated strains showing wide-spectrum QQ activity, genomic DNA was extracted using a Wizard DNA purification Kit (Promega, Madison, WI, USA), and the bacterial 16S rRNA gene was amplified using the universal primers 96 bfm (5′-GAGTTTGATYHTGGCTCAG-3′) and 1152 uR (5′-ACGGHTACCTTGTTACGACTT-3′) [59]. PCR were carried out under the following standard conditions: initial step of 96 °C for 2 min followed by 35 cycles at 95 °C for 1 min, 53 °C for 30 s, and 72 °C for 2 min. The 16S rRNA sequences were identified using the web-based tool EzTaxon [60].

### 2.3. Quorum-Sensing Activity Assay

The marine bacterial collection (605 strains) was screened for the strains' capability to activate the AHL biosensor *Agrobacterium tumefaciens* NTL4 [61,62]. The strains were cultured in microtiter plates in 200 μL of Marine Broth (MB) for 48 h. The plates were centrifuged, and the supernatants were transferred to a new plate. The pH of the supernatants was checked to be less than pH 8 in order to avoid lactonolysis of the AHLs produced at high pH values [63]. The presence of AHLs after the incubation period was detected by adding 50 μL of a mixture of soft Agrobacterium (AB) medium [61] (0.2% agar) with 5-bromo-4-chloro-3-indolyl-β-D-galactopyroside (X-GAL, 80 µg/mL) and an overnight culture of *A. tumefaciens* NTL4 (1:5) on top of the supernatants in microtiter wells. The plates were incubated for 6–8 h at 30 °C, and the production of blue colour on the surface of the wells was checked. AB medium pH 6.5 plus the C6-HSL (10 μM) was used as control. *A. tumefaciens* NTL4 was cultured at 22 °C in LB or AB medium supplemented with 30 µg gentamycin/mL.

The capacity of the strains presenting wide-spectrum QQ activity to activate the sensor *A. tumefaciens* NTL4 was confirmed with a disk-diffusion agar plate assay [modified from 50] at different times. One mL of an overnight shaken culture of the biosensor was mixed with 4 mL of soft AB medium (0.8%) with X-GAL (80 µg/mL) to cover the AB agar plates. Once the plates had solidified, 10 µL of supernatant from the 24 h and 48 h cultures of the 12 wide-spectrum QQ strains was loaded in antibiogram disks and deposited on the plates. The plates were incubated at 22 °C for 24 h. PBS pH 6.5 plus C6-HSL was used as a control, and the presence of a blue induction halo around the disks was checked. The stock of C6-HSL was prepared in acetonitrile at a concentration 1 mg/mL. This stock solution was diluted in PBS to a final concentration of 10 μM and added to the disks. 

### 2.4. Quorum-Quenching Activity Assay

The QQ activity of the strains was tested using a solid microtiter plate assays carried out with the AHL biosensors *Chromobacterium violaceum* VIR07 [64] for C12-HSL and *C. violaceum* CV026 [65] for C6-HSL. Two hundred microliters of the 48 h cultures carried out in microtiter plates in MB were centrifuged, and the pellets were washed with phosphate buffered saline (PBS) pH 6.5 and resuspended in another 200 μL of the same buffer. These cell suspensions were used for live-cell AHL degradation assay by adding either C6-HSL or C12-HSL (10 µM in PBS, prepared from a concentrated stock—1 mg/mL in acetonitrile) and incubating for 24 h at 22 °C. The presence of AHLs after the incubation period was detected by adding 50 μL of a mixture of soft Luria–Bertani (LB) (0.2% agar) and an overnight culture of the corresponding *C. violaceum* biosensor on top of the cell suspension in microtiter wells. The plates were incubated for 24 h at 30 °C, and the production of violacein was observed. PBS pH 6.5 plus the corresponding AHL (10 μM) was used as a control [66]. The same assay was used to check the capacity to degrade the oxo-substituted AHLs OC6-HSL and OC12-HSL. In order to detect false positives derived from the inhibition of the growth of the *C. violaceum* biosensors, all positive strains were re-isolated, and their activity confirmed with a Petri dish solid bioassay as described previously [47]. The Petri dish bioassays allow distinguishing growth inhibition (presence of a transparent halo around the well) from QS inhibition. In the *C. violaceum* assay, because of the high concentration of added exogenous AHL (10 μM), the absence or reduction of the violacein halo was considered indicative of enzymatic degradation. In this assay, the presence of an inhibitory substance is generally visualized as a clear, not transparent, halo around the well, surrounded by an external halo of violacein. Nevertheless, the presence of a very high amount of a QS inhibitor counteracting the action of the exogenous AHL could not be fully excluded. The biosensor strains were maintained in LB plates supplemented with kanamycin (30 µg/mL).

### 2.5. Characterization of AHL-Degradation Activity

Crude cell extracts (CCE) were obtained as previously reported [67]. Briefly, the cell biomass was obtained by centrifugation, resuspended in PBS, sonicated on ice, and centrifuged again. The cell extract obtained was filtered through a 0.20 μm filter and stored at 4 °C. To determine the minimal active concentration (MAC) of the CCEs, C6-HSL (10 μM) was exposed to different dilutions of CCEs, in PBS pH 6.5. The mixture was incubated for 24 h at 22 °C, and the presence of a signal was detected using the *C. violaceum* CV026 Petri dish bioassay. The control wells were filled with sterile PBS pH 6.5 plus AHL (10 μM).

### 2.6. Metagenomic Samples

Eight samples (Med-OCT2015-15 m, Med-OCT2015-30 m, Med-OCT2015-45 m, Med-OCT2015-60 m, Med-OCT2015-75 m, Med-OCT2015-90 m, Med-OCT2015-1000 m, and Med-OCT2015-2000 m) [55,56] from different depths were taken for metagenomic analyses on October 15, 2015 at a single sampling site in the Western Mediterranean (37.35361° N, 0.286194° W). Six samples were obtained from the uppermost 100 m at 15 m intervals using a hose attached to a CTD (Seabird). Another two samples from the depths of 1000 m and 2000 m, were taken the next day (October 16) in two casts (100 L each) using the CTD rosette.

All seawater samples were sequentially filtered on board through 20, 5, and 0.22 μm pore size polycarbonate filters (Millipore, Billerica, MA, USA). All filters were immediately frozen on dry ice and stored at −80 °C until processing. DNA extraction was performed from the 0.22 and 5 μm filters, as previously described [68]. The metagenomes were sequenced using Illumina Hiseq-4000 (150 bp, paired-end read) (Macrogen, Seoul, Korea). 

### 2.7. Metagenomic Analysis

The metagenomic analysis (read pre-processing, assembly and gene prediction and annotation) was carried out as previously described [56]. In brief, each metagenome was assembled independently using IDBA-UD [69]. The genes obtained on the assembled contigs were predicted using Prodigal [70] transfer RNA (tRNA), and the rRNA genes were predicted using tRNAscan-LE [71], ssu-align [72], and meta-rna [73]. A taxonomic and functional annotation was performed comparing the predicted protein sequences against NCBI NR, COG [74], and TIGFRAM [75] databases were performed using USEARCH6 [76]. USEARCH6 with an *e*-value <1e-5 was also used to identify potential 16S sequences from a subset of 10 million reads for each metagenome against RDP database [77]. These candidates were then aligned to archaeal, bacterial, and eukaryal 16S/18S rRNA HMM models [78], using ssu-align to identify true sequences [72], using a threshold of sequence identity ≥80% and alignment length ≥90 bp. In order to analyze the 16S rDNA diversity, identical sequences (99.9% of identity) from both datasets were removed using CD-HIT software [57]. The rest of the sequences were clustered to operational taxonomic units (OTUs) defined at 95% identity. Taxonomic affiliations of these clusters were assigned using the assigned Ribosomal Database Project (RDP) database [58].

Only AHL lactonase and AHL acylase genes with demonstrated activity were used within the QQ enzymes (Supplementary Material). The analysis of other QS-related genes, such as those for AHL synthases (*LuxI*, *AinS*, and *HdtS*) and AHL receptors (*LuxR* and *AinR*) as well as the gene for the synthase responsible for producing the AI-2 signal (*LuxS*), was carried out by aligning the metagenomic reads against the NR database using DIAMOND [79] (blastx option, top hit, ≥50% identity, ≥50% alignment length, *e*-value < 10^−5^). The abundance of these genes was normalized by the number of reads matching *rec*A + *rad*A sequences for each metagenome. The reads that gave hit to viral or eukaryal proteins were not taken into account. In order to analyse the relative abundance of the QQ enzymes in the water column, we applied the same sequence search for genes involved in the normal metabolism derived from marine bacteria, such as nitrogen (*amoC*, *amt*), phosphate, (*pstA*), sulfur (*dsrA*, *soxB*), and general oxidative metabolism (*dmdA*) [48,80].

### 2.8. Data Availability

The metagenomic datasets used in this study were submitted to NCBI SRA and are available under BioProjects accession number PRJNA352798 (Med-OCT2015-15 m, Med-OCT2015-30 m, Med-OCT2015-45 m, Med-OCT2015-60 m, Med-OCT2015-75 m, Med-OCT2015-90 m, Med-OCT2015-1000 m and MedOCT2015-2000 m).

### 2.9. Statistical Analysis

The effects of depth and culture media on the number of CFUs/mL was analysed with the non-parametric Mann–Whitney test at significance level *p* < 0.05, with IBM SPSS statisticsV20 program.

## 3. Results

### 3.1. Bacterial Growth and Isolation

**Figure 1:**
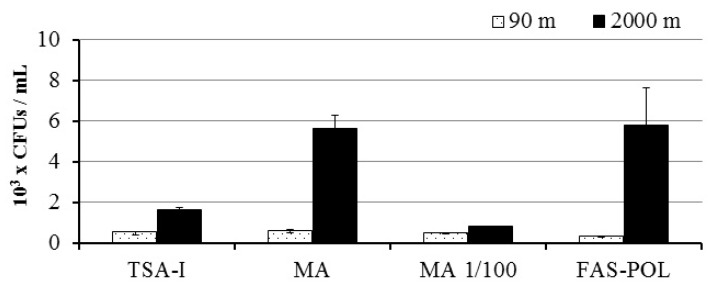
Cultivable bacteria concentration (colony forming units (CFU)/mL, average ± s.d., *n* = 5) obtained in the photic (90 m, white bars) and aphotic (2000 m, black bars) samples for the culture media TSA-1%NaCl (TSA-1), Marine Agar (MA), Marine agar diluted 1:100 in seawater (MA 1/100), and Filtered autoclaved sea-water enriched with polymers (FAS-POL).

The number of CFUs/mL was significantly higher in the 2000 m (0.8–5.8 × 10^3^ CFUs/mL) than in the 90 m water sample (0.2–0.6 × 10^3^ CFU/mL) for the different culture media tested (Mann–Whitney Test, *p* < 0.05), despite the isolation conditions excluded the retrieval of barophilic or psychrophilic strains from the deep-sea sample. The culture media did not affect the number of CFUs/mL obtained in the 90 m, photic sample (Figure 1, Mann–Whitney Test, *p* > 0.05). On the contrary, the CFUs obtained in MA and FAS-POL culture media were significantly higher in the sample from 2000 m, yielding three times more CFUs than the other media (Figure 1, Mann Whitney Test, *p* < 0.05). 

### 3.2. Taxonomic Diversity of the Cultivable Strains

**Figure 2:**
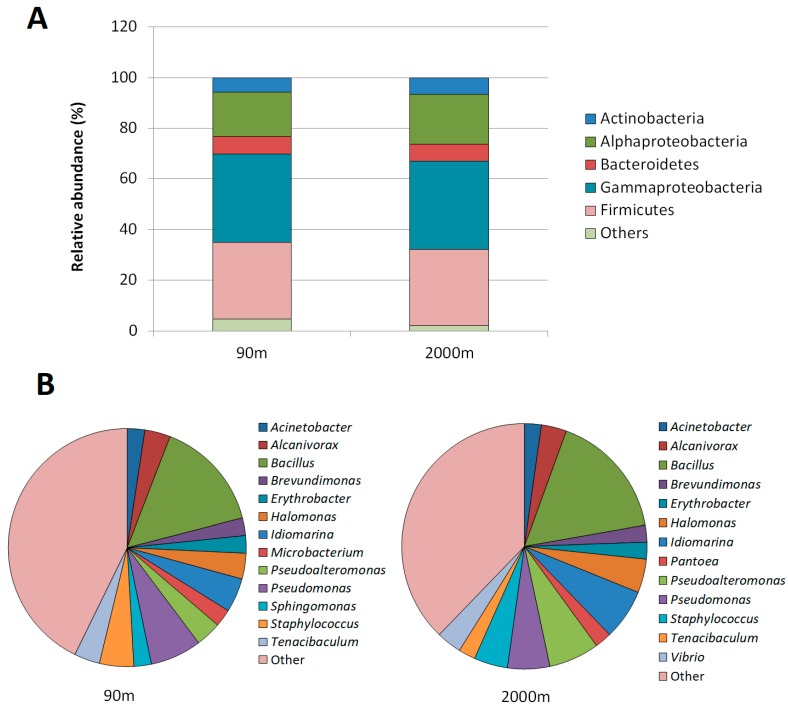
(**A**) Bacterial diversity in cultivable bacteria isolated from photic (90 m depth, *n* = 231) and aphotic (2000 m depth, *n* = 374) samples; (**B**) relative abundance of the 13 most abundant genera in 90 and 2000 m depth samples. The genera represented by a single isolate are grouped as “other”.

A total of 605 isolates, 231 from the 90 m and 374 from the 2000 m sample, were collected and screened for their capacity to activate a QS biosensor and for their QQ activity against AHLs (Supplementary Table S1). The most cultivable strains belonged to Gammaproteobacteria (34.88%), Firmicutes (30.95%), and Alphaproteobacteria (17.44%). Members of the Actinobacteria (6.84%) and Bacteroidetes (7.14%) were less represented (Figure 2A). Firmicutes were highly represented in the cultivable collection (30% in both samples), in comparison with the data obtained from the metagenomic analysis at the same depth (<1%) [56]. Gammaproteobacteria (35%) were also overrepresented in the 90 m sample in comparison with the metagenomic data (13.56%). The relative abundance at the family level showed similar profiles at both depths, except for *Vibrionaceae* and *Rhodobacteraceae* which were only identified at 2000 m (Figure 2A). However, when the OTUs were clustered at 95% of identity from both datasets to separate them at genus level, 56 and 82 genera could be identified at 90 and 2000 m, respectively, showing that there was a greater genetic diversity at 2000 m that could not be appreciated at higher taxomonic levels. In the future, the genome sequencing of these organisms could clarify and shed light on many of the differences between these two depths. Despite of this, the two collections of cultivable bacteria shared 11 of the 13 most abundant genera (Figure 2B). Surprisingly, *Bacillus* was the most abundant group among the cultivable bacteria, representing 15.74% and 16.6%, in the 90 and 2000 m samples, respectively. The genera *Microbacterium* (2.38%) and *Sphingomonas* (2.38%) were exclusively identified in the sample from the 90 m depth, meanwhile the genus *Pantoea* and *Vibrio* appeared only in the sample from 2000 m (Figure 2B). 

### 3.3. Quorum-Sensing and Quorum-Quenching Activities among Cultivable Bacteria 

**Figure 3:**
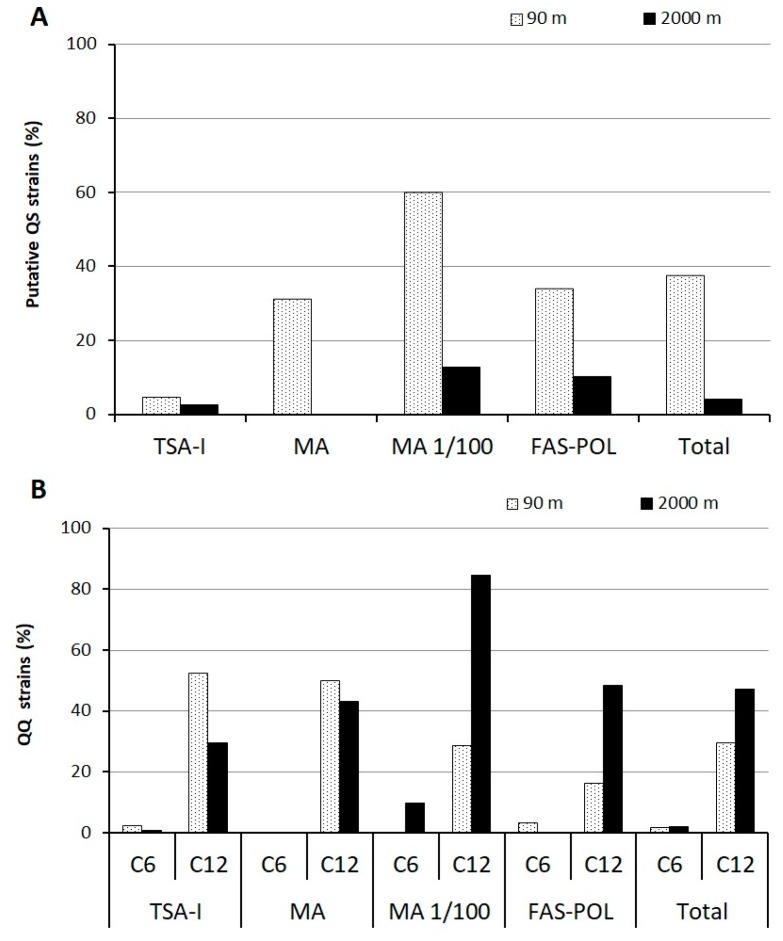
(**A**) Percentage of the isolated strains with the ability to activate the *N*-acyl homoserine lactone (AHL) sensor *Agrobacterium tumefaciens* NTL4 in the 90 m (white bars) and 2000 m (black bars) samples; (**B**) percentage of isolates with quorum-quenching (QQ) activity against C6 and C12-HSL isolated from each culture media in the 90 m (white bars) and 2000 m (black bars) samples, as confirmed by using the Petri dish solid assay with *Chromobacterium violaceum* CV026 and VIR07. Media used: tryptone soy agar 1% NaCl (TSA-1), marine agar (MA), diluted marine agar (MA 1/100), and filtered autoclaved seawater medium (FAS) supplemented with 0.5 g/L polymers: agarose, chitin and starch (FAS-POL).

The activation of the beta-galactosidase gene of the sensor, which suggests the presence of AHLs in the culture media, was relatively frequent among the 605 marine isolates (20.84%). The number of positives was higher in the samples from 90 m (37.66%) than in ones from 2000 m (4.01%) (Figure 3A). The oligotrophic MA 1/100 culture medium allowed the isolation of the higher percentage of strains with putative QS activity at both 90 (60%) and (12.67%) 2000 m. On the contrary, the strains isolated from TSA-I presented a lower capacity to activate the reporter (4.76 and 2.52% for 90 and 2000 m samples respectively).

The capacity of the marine bacterial collection to interfere with both long- (C12-HSL) and short-chain (C6-HSL) AHLs was first tested using a *C. violaceum*-based bioassay in 96-well microtiter plates. None of the QQ positives in the preliminary assay showed interference with the growth of the biosensors. The ability to quench the QS signal C12-HSL was observed in a large number of cultivable bacteria (38.24%). The average activity was higher in the 2000 m, aphotic sample (47.05%) than in the 90 m photic one (29.43%, Figure 3B). Among the 68 strains isolated from the 90 m depth with the capacity to quench C12-HSL, four strains, representing 1.73% of the total strains, were also able to eliminate the C6-HSL QS signal, and a similar percentage was obtained for the isolates from the 2000 m sample (2.12%). All the isolates with the ability to interfere with C6-HSL could also degrade C12-HSL, but no isolate was found that could only eliminate the activity of the short-chain AHL. The oligotrophic MA 1/100 culture medium allowed the isolation of the highest percentage of strains with QQ activity against C12-HSL (84.5%) in the 2000 m sample. On the contrary, in the 90 m sample, the rich culture media (TSA-I and MA) presented the highest percentages of QQ activity (50–52%). The most effective culture medium to isolate strains with QQ activity against the short-chain signalC6-HSL were the oligotrophic FAS-POL (3.22%) and MA 1/100 (9.85%) for the samples from 90 and 2000 m, respectively. 

### 3.4. Identification of the Wide-Spectrum Quorum-Quenching Strains and Characterization of Quorum-Quenching Activity 

The taxonomic label of the best hit is shown in Table 1 for the 12 strains presenting wide-spectrum QQ activity. The four marine strains selected from the 90 m sample belonged to Firmicutes (*Planomicrobium chinense*), Alphaproteobacteria (*Sphingopyxis alaskensis*, *Erythrobacter citreus*), and Actinobacteria (*Microbacterium schleiferi*). Among the eight strains from the deepest sample, one of them belonged to Betaproteobacteria (*Ralstonia pickettii*), six to Alphaproteobacteria (5 *Erythrobacter flavus* strains, *Citomicrobium* sp.), and one to Gammaproteobacteria (*Pantoea* sp.). Despite the high number of *Bacillus* sp. strains present in the collections, no *Bacillus* strain with wide-spectrum QQ activity was identified.

The isolate 2F1 may represent a new species within the genus *Ralstonia* because of the low identity with the known species of the genus (95.79%). The cell extracts of three isolates, 3A3 (*Microbacterium schleiferi)*, 4B7 (*Pantoea* sp.), and 4C3 (*Citromicrobium* sp.) did not present QQ activity against C6-HSL. The ability to interfere with oxo-substituted AHLs was also tested for the 12 selected strains (Table 1). Most of the marine strains were able to inactivate the oxo-substituted AHL, except for strain 4B7 (*Pantoea* sp.) that did not show QQ activity against any of the substituted AHLs tested, namely OC6 and OC12-HSL. In order to check the relative activity of the strains, the minimal active concentration (MAC, µg protein/mL) was calculated in the cell extracts. The marine isolates with the highest activity were 2E12 (*Sphingopyxis alaskensis*) and 2F1 (*Ralstonia* sp.), since their QQ activity was at least one order of magnitude higher than that of the other QQ strains (<20 µg/mL). 

Among the 12 strains, *Sphingopyxis alaskensis* 2E12 and *Erythrobacter citreus* 2G12 could activate the biosensor *A. tumefaciens* NTL4 using 24 h supernatants. This activity was maintained in 48 h supernatants only for *Sphingopyxis alaskensis* 2G12, but disappeared in *Erythrobacter citreus* 2G12. AHL-like activity was also detected in 48 h supernatants of *Citromicrobium* sp. 4C3. 

### 3.5. Quorum-Sensing and Quorum-Quenching Genes in Metagenomic Samples

**Figure 4:**
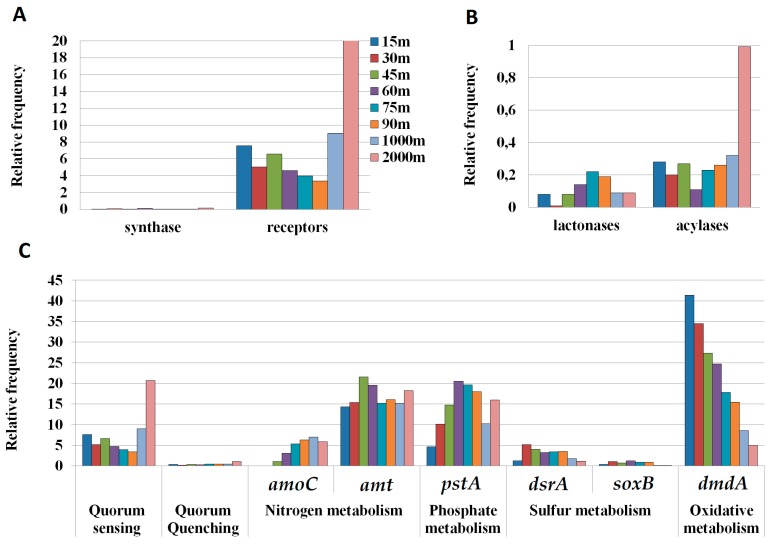
(**A**) Relative frequencies of AHL synthases and AHL receptors; (**B**) relative frequencies of QQ genes including lactonases and acylases; (**C**) relative frequencies of AHL-based QS and QQ sequences in comparison to other genes involved in nitrogen (*amoC*, *amt*), phosphate (*pstA*), and sulfur (*dsrA*, *soxB*) acquisition, as well as in oxidative metabolism (*dmdA*). The data was normalized in function of the abundance of the *recA* and *radA* housekeeping genes.

A search for QS-related genes, AHL synthases (*LuxI*, *AinS* and *HdtS*) and AHL receptors (*LuxR* and *AinR*) as well as the synthase responsible to produce the AI-2 signal (*LuxS*), was carried out in the metagenomic samples from the depth profile. The relative abundance of QS-related genes seemed to slightly decrease with depth in the first 100 m (7.59–3.41, Figure 4A). However, a high relative abundance of AHL receptors was observed in the two aphotic zone samples, the 1000 m (9.02) and especially the 2000 m samples (20.52). Remarkably, an extremely low presence of AHL synthases was found in the metagenomic analysis along the whole water column in comparison with the receptors frequency (Figure 4A). Surprisingly, no AI-2 synthase *LuxS* homologs were detected in any of the tested samples, despite the presence of *Vibrio* in the bacterial cultivable collection (3.5%).

We searched for the presence of AHL-lactonases and acylases genes, using sequences with proved activity, in the same metagenomic samples. Among the QQ genes, AHL acylases were more frequent than AHL lactonases in most metagenomic samples (0.99, Figure 4B). The analysis showed a clear increase of the frequency of acylases at 2000 m (0.99, Figure 4C), in the same sample that yielded the highest number of LuxR homologues (Figure 4A). The increase in total QQ sequences at 2000 m was due to an increase in acylase sequences, since the relative abundance of acylase sequences was similar in the first 1000 m (0.11–0.32) but increased sharply at 2000 m (0.99) (Figure 4B). In contrast, the lactonase sequences reached the highest presence in the 75 m sample (0.22) and decreased in the samples from greater depths. 

In order to analyze the relative abundance of the QQ enzymes in the water column, we applied the same sequence search for genes involved in the normal metabolism of marine bacteria, such as those involved in nitrogen (*amo*C, *amt*), phosphate, (*pst*A), sulfur (*dsr*A, *sox*B), and general oxidative metabolism (*dmd*A) (Figure 4C) [43,65]. The relative frequency of QS- and QQ-related genes was smaller than the frequency of the genes related to the common uptake transporters for ammonia (*amt*) or phosphate (*pst*A). However, the frequency was in the same range of those of *amo*C and of genes related to sulfur metabolism (*dsr*A, *sox*B). 

## 4. Discussion

Understanding how bacteria interact with each other is essential for predicting their role in the marine microbial environment and their potential impacts on marine ecology [14]. The ability of microorganisms to modulate the behavior of an entire population through the coordination and regulation of gene expression is viewed as an evolutionary adaptation to survive in a changing environment [81]. Despite the QS and QQ inhibition processes were discovered in the marine environment [35,82], little attention has been paid to the ecological significance of QS and QQ mechanisms in seawater, and this issue has been the center of controversy [13,14]. Increasing evidence points to an important role of AHL-mediated QS in different marine environments [14], and the frequent presence of QQ activity among marine bacteria isolated from several niche habitats further supports the ecological significance of AHL-dependent QS in the highly competitive marine environment. High QQ activity may be interpreted as the result of either an adaptative trait of strains presenting this QQ capacity in a situation in which QS processes increase bacterial species fitness, or a situation in which the concentration of AHL molecules is high enough to constitute a significant source of carbon. In previous works, a high number of strains with QQ activity (2–46%) were isolated from different marine environments [46,47,48,50,51,52,53,54]. However, the majority of the studies only used cultivation-dependent techniques, introducing an important bias in the evaluation of the significance of this activity. In the present case, we have compared the potential AHL production and degradation activity in bacteria isolated from the Mediterranean Sea at different depths through biosensor-based screening methods, with the number of putative QS and QQ genes found in metagenomes obtained from the same marine samples. This simultaneous study allowed us to obtain a more complete assessment of the possible prevalence of these processes, avoiding the limitations of the culture-derived and metagenomic search methodologies. While a cultivable bacteria analysis only allows to evaluate a small percentage of the total population, metagenomic analysis results do not take into account the possible promiscuity of substrates in the retrieved QQ sequences and the actual expression of these genes under environmental conditions. 

A higher cultivable bacterial density was observed in the aphotic, 2000 m depth sample (up to 5.64 × 10^3^ CFU/mL) than in the 90 m, photic sample (up to 0.6 × 10^3^ CFU/mL), despite no specific culture strategy was applied to retrieve psychrophilic or barophilic strains. The culture medium used for the estimation is an important factor, as demonstrated by the results obtained in the 2000 m depth sample (Figure 1) and in previous studies in the Atlantic Sea [42,48]. The opposite trend was observed in the counts of heterotrophic bacteria obtained by flow cytometry that yielded three times more bacteria at 90 than at 2000 m depth [56]. This discrepancy may reflect a higher density of cultivable, copiotrophic bacteria in the aphotic sample, which is further supported by the higher counts obtained in carbon-rich AM and FAS-POL culture media for this sample. Higher total P and N were available at 2000 m (0.5 μM and 8.6 μM respectively) in comparison to the sample from 90 m (0.25 μM and 6.9 μM), which may also explain the higher CFUs/mL retrieved in the aphotic zone sample. On the contrary, lower TOC was available in the 2000 m aphotic sample (0.94 mg C/L against 1.35 mg C/L), which could generate a highly competitive, nutrient-limited environment in the aphotic sample. 

While important differences in the community structure were found associated with depth in the metagenomics analysis [56], the diversity of the isolated strains was very similar in the samples collected at 90 and 2000 m, with most strains belonging to the Proteobacteria and Firmicutes phyla. This difference is not unexpected, as only about 1% of the total marine microbes can be isolated on artificial media, while the vast majority remains uncultivable [83]. Moreover, metagenomes avoid the bias imposed by culturing techniques, which prioritizes the growth of *r*-strategies microbes that are expected to dominate the cultivable population. Proteobacteria were the most prevalent lineage in both the metagenomic dataset (43%) and the cultivable bacteria collection (52.38%), but the percentage of readings associated with Firmicutes was less than 1% in any of the depths in the metagenomic diversity, while this class represented around 30% of the cultivable bacteria. Furthermore, while the Alphaproteobacteria dominate the bacterial population in the Mediterranean Sea, decreasing in abundance with depth [56,84], in the cultivable collections this group represented only 18% with no difference between the photic and aphotic samples. 

The data obtained from the Mediterranean Sea bacterial collections obtained from 90 and 2000 m depths showed a high percentage of strains with the ability to activate the AHL-reporter *A. tumefaciens* NTL4. Although the culture conditions could strongly affect QS signal production, and the detection of AHL-like activity in the cultures does not guarantee that the signals are produced under environmental conditions [5], the results suggest that AHL-mediated QS systems could be a common coordination mechanism in marine bacterial communities. It should be noted that the microtiter-based screening method does not allow high growth rates, and, therefore, the number of AHL producers could be underestimated, at least under laboratory conditions, as demonstrated for the identification of additional positive strains when cultured in higher-volume, shaken cultures. A higher abundance of putative AHL producers was found in the photic, 90 m sample (37.66%) in comparison with the deep-sea sample (4.01%). Lower P availability and higher bacterial density may justify the higher relevance of QS mechanisms at 90 m, since AHLs have been proposed to mediate and coordinate the mechanisms of processing and acquisition of P [29]. A prevalence of QS systems has been demonstrated in nutrient-deficient conditions [85], which could also be correlated with the higher putative QS activity found under laboratory conditions among isolates obtained with the oligotrophic culture media MA 1/100 and FAS-POL. 

The screening for QQ activity showed that 38.24% of the strains were able to intercept the long-chain QS signal C12-HSL. Although AHL-like activity was also detected in the supernatants from some of these QQ strains, it is unlikely that the observed inhibitory effect can simply be ascribed to the inhibitory effect of non-cognate AHLs, since the QQ activity is still present in the cell extracts, and the concentration of the signal would be too low to counteract the high AHL concentration used in the QQ screening assays (10 µM). In the same way, and although we cannot fully disregard the fact that some of the strains with QQ activity were producing a high concentration of a QS inhibitory substance, the high AHL concentration used in the bioassays supports the fact that the observed QQ activity should be mainly of enzymatic nature. Additional biochemical analysis should be performed to confirm this hypothesis [86]. The high percentage of cultivable strains identified in both photic and aphotic samples with this capacity indicates that QQ may be a common activity in the Mediterranean Sea, which is generally characterized by oligotrophy [87]. Opposite to the putative QS activity, QQ activity against C12-HSL was almost 1.63-fold at 2000 m (47.05%) in comparison with the sample from 90 m (29.43%). This abundance correlates with lower POC availability in the deeper sample and may therefore indicate a highly competitive environment in which the bacterial population capable of using the long-chain AHL as a source of carbon may have an ecological advantage. Although pH, salinity, pressure, and intensity of the UV irradiation of the seawater have a significant influence on the production, stability, and perception of the QS signal molecules [13,24,88,89], the differences in the physicochemical parameters between the two samples [56] do not justify the differences in QS and QQ activity.

A large difference was observed between the ability of the strains to degrade the long-chain signal C12-HSL (38.24%) or the short-chain signal C6-HSL (1.93%). Unlike the degradation of C12-HSL, the percentage of strains being able to degrade C6-HSL was similar in both samples (1.73 and 2.12%). This low prevalence of strains with wide-spectrum QQ activity is similar to the percentage reported for soil samples against the C6-HSL (2.5%) [43]. The more diffused degradation of long-chain AHLs signals among marine bacteria could be related to the self-degradation process that the lactone ring suffers at the high pH of seawater, which occurs faster for the short-chain AHLs [53]; therefore, specific enzymatic degradation would be more necessary for the long-chain molecules. This data would also support the idea that QS signals can be used as an additional carbon and energy source under carbon-limitation conditions, since the degradation of a long-chain signal such as C12-HSL could contribute more efficiently than that of the shorter ones to the metabolic budget of the cells. 

The wide-spectrum QQ strains identified in this study belong to Alphaproteobacteria (eight strains), Betaproteobacteria (one strain), Gammaproteobacteria (one strain), Actinobacteria (one strain), and Firmicutes (one strain). Additionally, a novel species belonging to the genus *Ralstonia* was identified (strain 2F1). *Microbacterium schleiferi* and *Ralstonia* sp., isolated from 90 m and 2000 m depth, respectively, belong to a genus with previously reported QQ activity [89,90,91,92]. On the contrary, this is the first report on the ability to intercept AHL-mediated communication of members from the genus *Planomicrobium*, *Sphingopyxis*, *Erythrobacter*, *Pantoea*, and *Citromicrobium*. As previously reported [46,47,48,50], the taxonomic diversity found in the marine bacteria with wide-spectrum QQ activity is much higher than in soil and plant samples, where mainly *Bacillus* strains were identified [43,44]. Surprisingly, in our case, despite the high number of *Bacillus* strains present in the collection (15%), none of them showed wide-spectrum QQ activity. Most of the strains with QQ activity isolated in this study belong to the Alphaproteobacteria, including representatives of the genus *Citromicrobium*, *Erythobacter*, and *Sphyngopyxis*. Alphaproteobacteria represent more than 15% of the cultivable bacteria analyzed and 25% of the metagenomic diversity (Figure 2) [56]. Although the abundance of *Erythrobacter* strains in the cultivable collection was similar in both samples (2.30%), surprisingly, those from 2000 m depth presented QQ activity more frequently (five strains against one). Differences in the QQ activity among species of the same genus have been already reported [66], and, in this case, the higher activity among isolates from the deeper sample may further support an adaptative value of this activity in carbon-limited environments. Regarding the activity present in the crude cell extract (CCE) of the wide-spectrum QQ strains, strains 2E12 (*Sphingopyxis alaskensis*) and 2F1 (*Ralstonia* sp.) presented the strongest QQ activity, since their MAC were 18.67 and 17.6 μg CCE protein/mL, respectively. This value is similar to the MAC reported for *Tenacibaculum* sp. 20J, a bacterium with high QQ activity [66,67]. Further characterization of the QQ activity of these strains in order to confirm their QQ enzymatic activity with additional biochemical analyses [86,93] will certainly contribute to the search for novel anti-pathogenic compounds with potential applications to control bacterial infections in plants [42], aquaculture [31,51,67], and more recently in biomedicine [94].

We have observed a clear increase in the presence of the QS and QQ putative sequences with the sample depth. The relevance of these genes is especially evident in the aphotic samples, in which the abundance of the QS and QQ sequences was higher than that of sequences related to sulfur (*dsrA* and *soxB*), nitrogen (*amoC*), and oxidative metabolism (*dmdA*). The similar frequency of QQ enzymes in relation with other important processes might suggest a potential relevance of these processes in the sea, mainly in deeper waters. A further analysis would be necessary in order to asses if QS and QQ genes are actually expressed in the marine environment.

While the metagenomic data are consistent with the higher QQ activity found among cultivable bacteria in deeper samples, an important discrepancy was found between the higher presence of QS-related genes in the 2000 m sample and the lower putative QS activity in the cultivable bacteria obtained from the same sample. As explained before, the bias derived from the use of culture conditions not resembling the natural marine environment may be the cause of this discrepancy. A recent study on four different *Escherichia coli* strains showed that the 47% of the variance in expression levels is dependent primarily on the environment (medium-dependent genes) [95]. Therefore, the present in vitro experiments indicate the ability of a high percentage of the marine strains to activate AHL sensors and quench the AHL molecules; however, further environmental in vivo studies are needed to confirm and complete our knowledge regarding the role and relevance of AHL-mediated QS in the marine environment.

The higher percentage of cultivable strains with AHL-type QS and QQ activity strongly supports the significance of this activity in marine samples, however, the results derived from the metagenomics analysis should be interpreted with caution. First, the detection of sequences with high similarities does not ensure the conservation of the functionality or the expression of the gene under specific environmental conditions. Secondly, since other possible biochemical activities could be attributed to the QQ enzymes, the high prevalence cannot be unequivocally related to AHL degradation. In this sense, the Mediterranean metagenomes analyzed yielded a higher number of acylases than lactonases (Figure 4), despite the widest diversity of AHL lactonases identified so far [4,39]. A higher prevalence of acylases has also been reported previously in the GOS sequences [48], even though at that time the number of known lactonases was much lower. Typically, AHL acylases are considered more substrate-specific than lactonases and have been defined as exclusive AHL degraders. However, the recent identification of acylases with a broad specificity which can also degrade penicillin G [96,97], has added complexity to the metabolic role of AHL acylases. This promiscuity of substrates could partially explain this higher frequency and question the exclusive activity of these enzymes in the degradation of AHL-type QS signals. Therefore, and despite the data obtained from the metagenomic analysis seem to confirm the highest prevalence of QQ activities in deeper samples, this result should be interpreted with caution until the QQ activity of some of the retrieved sequences can be confirmed. 

In this study, we have found that the presence of the three types of AHL synthases was very low in the entire water column. The study of metagenomic databases from the Atlantic, Pacific, and Indian Oceans [33] demonstrated that the QS genes *LuxI*, *AinS*, and especially *HdtS* are present in a much wider diversity of marine bacteria than previously suspected from cultivable bacteria, and they were more frequently identified (0.02–14.8) in comparison to the present study (0.01–0.17). As described here for the variability in the QQ activity, differences in nutrient availability or physicochemical conditions may be responsible for these differences among sampling sites, although differences in the methodology applied cannot be disregarded as a significant source of variation. AHL receptors were much more common than AHL synthases in the Mediterranean samples, especially at 2000 m depth (Figure 4A), correlating with a higher abundance of QQ activity and genes. In agreement with this unbalance between QS synthases and receptors, a previous study showed that among the 68 genomes of Proteobacteria compared, 45 contained orphan LuxR homologs but no additional LuxI homologs, and 66% showed more LuxR than LuxI homologs [98]. This unbalance can be attributed to a large prevalence of orphan LuxR receptors in the bacterial population. The microorganisms with an orphan LuxR do not regulate directly the AHL synthesis, but they can participate in the cell-to-cell bacterial communication. This supports the idea that the ability to “sense” other bacteria is more efficient than the capacity to “talk” to them, since the AHL-production is highly energy costly. Moreover, other molecules, such as cinnamoyl-HSL, *p*-coumaroyl-HSL, α-pyrones, and dialkylresorcinols, have been reported to activate LuxR-type receptors [11]. Nevertheless, in the view of the large unbalance between the number of synthases and receptors identified in the Mediterranean metagenomes, the hypothesis of the existence of other yet unknown AHL synthases cannot be excluded. 

Surprisingly, and despite *Vibrio* sp. representing a 3.5% of the bacterial population in the analyzed samples, no LuxS homologs could be identified in the entire water column, indicating that the AHL-mediated QS processes are more abundant than the AI-2 signaling pathway in the marine environment. In coherence with this result, LuxS homologues could be identified in only 3 of the 13 sites included in the GOS database, with a normalized frequency of 0.7 [33]. This data on the prevalence of the *luxS* gene contrasts with the 653 sequences (14.8) affiliated to HdtS in the same study, confirming that the AHL-mediated QS processes are more frequent than the AI-2 signaling in the marine environment. 

The high percentage of isolated strains with the ability to activate AHL biosensor and to disrupt long-chain AHL QS signals in the Mediterranean Sea, combined with the high frequency of QQ and QS genes that correlate with lower C availability, indicates that QS and QQ could be usual strategies in this marine habitat that may confer a competitive advantage in oligotrophic conditions. Furthermore, the analysis of the metagenomic data indicates that QS and QQ processes have an important role in deep marine habitats. On site, ecological studies on QS and QQ activity and the identification of more QS- and QQ-related sequences in the marine habitats would probably provide key knowledge to further elucidate the ecological importance of AHL-mediated QS and QQ processes in the marine environment.

## Figures and Tables

**Table 1:** Identification and characterization of the marine isolates showing wide Quorum-Quenching (QQ) activity. The presence of QQ activity in the cell extracts and the minimal active concentration of extract (MAC, µg protein/mL cell extract) needed to fully eliminate the activity of 10 µM of C6-HSL was also investigated.

				Live Cell				Cell Extract		MAC C6-HSL (µg Protein/mL Cell Extract)
	Strain	Closest Cultivated Bacteria	% ID at 16S rRNA Gene Locus	C6-HSL	OC6-HSL	C12-HSL	OC12-HSL	C6-HSL	C12-HSL	
90 m	1F1	*Planomicrobium chinense*	99.93	+	+	+	+	+	+	104.7
	2E12	*Sphingopyxis alaskensis*	99.93	+	+	+	+	+	+	18.67
	2G12	*Erythrobacter citreus*	99.04	+	+	+	+	+	+	1918
	3A3	*Microbacterium schleiferi*	99.71	+	+	+	+	-	+	nd
2000 m	2F1	*Ralstonia pickettii*	95.79	+	+	±	+	+	+	17.6
	3G7	*Erythrobacter flavus*	99.34	+	+	+	+	+	+	786
	4B4	*Erythrobacter flavus*	99.34	+	+	+	+	+	+	216
	4B7	*Pantoea* sp.	96.01	+	-	+	-	-	-	nd
	4B9	*Erythrobacter flavus*	99.71	+	+	+	+	+	+	961
	4B10	*Erythrobacter flavus*	99.49	+	+	+	+	+	+	223.9
	4B12	*Erythrobacter flavus*	99.49	+	+	+	+	+	+	965
	4C3	*Citromicrobium* sp.	98.33	+	+	+	+	-	+	nd

Nd: Not determined.

## Supplementary Materials

The following are available online at http://www.mdpi.com/2073-4425/9/2/100/s1, Table S1: Total isolated strains from different samples and culture media, showing the number and percentage of strains with putative QS and QQ activity against C6- and C12-HSL obtained using the solid plate *C. violaceum* assay. Media used: tryptone soy agar 1% NaCl (TSA-1), marine agar (MA), diluted marine agar (MA 1/100), and filtered autoclaved seawater medium (FAS) supplemented with 0.5 g/L polymers: agarose, chitin, and starch (FAS-POL).
