# Supplementary material for: High Prevalence of Quorum-Sensing and Quorum-Quenching Activity among Cultivable Bacteria and Metagenomic Sequences in the Mediterranean Sea
Source: Genes (Basel). 2018 Feb 16;9(2):100. doi: 10.3390/genes9020100 (PMC5852596; doi:10.3390/genes9020100)
Supplement: Supplementary file 1 [file genes-09-00100-s001.docx]

Supplementary Material

High prevalence of quorum sensing and quorum quenching activity among cultivable bacteria and metagenomic sequences in the Mediterranean Sea

**Table S1.** Total isolated strains from different samples and culture media, showing the number and percentage of strains with putative QS and QQ activity against C6- and C12-HSL obtained using the solid plate *C. violaceum* assay. Media used: tryptone soy agar 1% NaCl (TSA-1), marine agar (MA), diluted marine agar (MA 1/100), and filtered autoclaved seawater medium (FAS) supplemented with 0.5 g/L polymers: agarose, chitin, and starch (FAS-POL).

|  | | Nº Isolates | Putative QS strains | %QS | QQ strains | | %QQ | |
| --- | --- | --- | --- | --- | --- | --- | --- | --- |
|  |  |  |  |  | C6-HSL | C12-HSL | C6-HSL | C12-HSL |
| 90 m | TSA-I | 42 | 2 | 4.76 | 1 | 22 | 2.38 | 52.38 |
|  | MA | 16 | 5 | 31.25 | 0 | 8 | 0 | 50.00 |
|  | MA 1/100 | 80 | 48 | 60.00 | 0 | 23 | 0 | 28.75 |
|  | FAS-POL | 93 | 32 | 34.40 | 3 | 15 | 3.22 | 16.12 |
|  | Total | 231 | 87 | 37.66 | 4 | 68 | 1.73 | 29.43 |
| 2000 m | TSA-I | 119 | 3 | 2.52 | 1 | 35 | 0.84 | 29.41 |
|  | MA | 155 | 0 | 0 | 0 | 67 | 0 | 43.22 |
|  | MA 1/100 | 71 | 9 | 12.67 | 7 | 60 | 9.85 | 84.50 |
|  | FAS-POL | 29 | 3 | 10.34 | 0 | 14 | 0 | 48.27 |
|  | Total | 374 | 15 | 4.01 | 8 | 176 | 2.12 | 47.05 |

**Quorum quenching enzymes sequences used in metagenomic searches:**

**Lactonases sequences:**

>AB197037.1 Mesorhizobium loti gene for 4-pyridoxolactonase, complete cds

>AB513359.2 Microbacterium testaceum aiiM genes for acylhomoserine lactonase, hypothetical proteins, partial and complete cds

>AB648919.2 Solibacillus silvestris ahlS gene for N-acylhomoserine lactonase, complete cds, strain: StLB098

>AB935247.1 Thermaerobacter nagasakiensis aiiT gene for acylhomoserine lactonase, complete cds, strain: JCM 11223

>AE016958.1:4075266-4076243 Mycobacterium avium subsp. paratuberculosis str. k10, complete genome (MCP)

>AF196486.1 Bacillus sp. 240B1 putative metallohydrolase (aiiA) gene, complete cds

>AF525800.1 Arthrobacter sp. IBN110 acyl homoserine lactone degrading enzyme (ahlD) gene, complete cds

>AF539592.1 Homo sapiens paraoxonase 1 (PON1) gene, complete cds

>AJ505742.1 Uncultured Bacillus sp. aii2 gene for N-acylhomoserine lactone lactonase

>AY052389.1:3565-4356 Agrobacterium tumefaciens AttM/AiiB (attM/aiiB) genes, complete cds

>AY210982.1 Homo sapiens paraoxonase 2 (PON2) gene, complete cds

>AY222324.1 Klebsiella pneumoniae AhlK (ahlK) gene, complete cds

>AY775568.1 Sulfolobus solfataricus strain MT4 phosphotriesterase-like lactonase gene, complete cds

>AY805220.1 Homo sapiens paraoxonase 3 (PON3) gene, complete cds

>BA000043.1:1545388-1546368 Geobacillus kaustophilus HTA426 DNA, complete genome

>CP002529.1:2209310-2210254 Vulcanisaeta moutnovskia 768-28, complete genome (VmoLac)

>CP016540.2:322503-323573 Planococcus sp. L10.15, complete genome (AidP)

>CP020592.1:1103067-1103978 Acinetobacter baumannii strain USA2, complete genome

>CP023170.1:273256-274224 Mycobacterium tuberculosis strain C3 (PPH)

>EF530726.1 Uncultured bacterium Bio1 BpiB01 (bpiB01) gene, complete cds

>EF530732.1 Uncultured bacterium Bio7 BpiB04 (bpiB04) gene, complete cds; and unknown genes

>EF530733.1 Uncultured bacterium Bio8 BpiB05 (bpiB05) gene, complete cds; and unknown gene

>EF655902.1:236-901 Uncultured Acidobacteria bacterium cosmid p2H8, complete sequence (QlcA)

>gb|AY541692.1|:1092-2063 Rhodococcus erythropolis acyl-CoA synthetase-like protein gene, partial cds; QsdA (qsdA) gene, complete cds; AhlR (ahlR) gene, partial cds; and unknown gene

>gb|GQ849010.1|:819-1634 Ochrobactrum sp. T63 peptidyl-tRNA hydrolase domain protein, alpha/beta hydrolase fold protein (aidH), pyruvate dehydrogenase, pantothenate kinase, and phosphoribosyl-ATP pyrophosphohydrolase genes, complete cds

>gi|394557574:2642-3634 Chryseobacterium sp. StRB126 aidC genes

>HM750248.1 Bacillus sp. AI96 N-acyl homoserine lactonase (aiiA) gene, complete cds

>JX392407.1 Pseudoalteromonas byunsanensis strain 1A01261 RND-type efflux transporter protein gene, partial cds

>KF709655.1 Enterobacter asburiae strain PT39 AHL-lactonase (aiiA) gene, partial cds

>KF768740.1 Enterobacter asburiae strain VT65 AHL-lactonase (aiiA) gene, partial cds

>KF768741.1 Enterobacter aerogenes strain VT66 AHL-lactonase (aiiA) gene, partial cd

>KF768742.1 Enterobacter ludwigii strain VT70 AHL-lactonase (aiiA) gene, partial cds

>KJ756328.1 Muricauda olearia strain Th120 metallo-lactamase family protein (murol1831) gene, complete cds

>KP836243.1 Enterobacter sp. CS25 AHL-lactonase (aiiA) gene, partial cds

>KR011986.1 Enterobacter sp. CS9 AHL-lactonase (aiiA) gene, partial cds

>KR049073.1 Enterobacter ludwigii strain CS16 AHL-lactonase (aiiA) gene, partial cds

>KR049074.1 Enterobacter aerogenes strain CS28 AHL-lactonase (aiiA) gene, partial cds

>KR232934.1 Tenacibaculum sp. 20J N-acyl homoserine lactonase gene, complete cds

>KR232935.1 Tenacibaculum discolor strain DSM 18842 N-acyl homoserine lactonase gene, complete cds

>KR232936.1 Tenacibaculum gallaicum strain CECT 7122 N-acyl homoserine lactonase gene, complete cds

>KR232937.1 Tenacibaculum soleae strain CECT 7292 N-acyl homoserine lactonase gene, complete cds

>KR232938.1 Tenacibaculum maritimum strain NCIMB 2154 N-acyl homoserine lactonase gene, complete cds

>KR232939.1 Tenacibaculum lutimaris strain DSM 16505 N-acyl homoserine lactonase gene, complete cds

>KR232940.1 Tenacibaculum aestuarii strain JCM 13491 N-acyl homoserine lactonase gene, complete cds

>KU219945.1 Lysinibacillus sp. Gs50 AHL lactonase (adeH) gene, complete cds

>LC155972.1 Sphingomonas ursincola gene for acylhomoserine lactonase and 3-isopropylmalate dehydratase small subunit, complete cds, strain: A1

>LC155973.1 Sphingomonas ursincola gene for acylhomoserine lactonase and 3-isopropylmalate dehydratase small subunit, complete cds, strain: SF1

>LC155974.1 Sphingomonas ursincola gene for acylhomoserine lactonase and 3-isopropylmalate dehydratase small subunit, complete cds, strain: SF8

>NC_012586.1:1603564-1604499 Sinorhizobium fredii NGR234 plasmid pNGR234b, complete sequence

**Acylase sequences:**

>AB306517.1 Shewanella sp. MIB015 aac gene for acy-homoserine lactone acylase, complete cds

>AF529198.1 Ralstonia sp. XJ12B acy-homoserine lactone acylase (aac) gene, complete cds

>AP013070.1:3316676-3318355 Pseudomonas putida NBRC 14164 DNA, complete genome (PvdQ gene)

>AY561759.1 Streptomyces sp. M664 N-acylhomoserine lactone-degrading acylase (ahlM) gene, complete cds

>BA000019.2:c4733973-4731430 Nostoc sp. PCC 7120 DNA, complete genome (AiiC)

>gi|28376388:1234-3618 Ralstonia sp. XJ12B acy-homoserine lactone acylase (aac) gene, complete

>gi|512381003:1211458-1213899 Pseudomonas putida NBRC 14164 DNA, QuiP gene

>gi|668235478:2596-4068 Acinetobacter sp. Ooi24 genes for endoribonuclease L-PSP, FMN reductase, AraC family transcriptional regulator, acylhomoserine lactone acylase AmiE, IS4 family transposase ORF 1, IS4 family transposase ORF 2, ssDNA-specific exonuclease RecJ, partial and complete CDS

>GU581165.1 Ochrobactrum sp. A44 AHL acylase (aiiO) gene, complete cds

>M15418.1 K.citrophila pac gene encoding penicillin acylase, complete cds

>NC_003318.1:c224620-223223 Brucella melitensis 16M chromosome II, complete sequence (Aibp)

>NC_007005.1:2285834-2288173 Pseudomonas syringae pv. syringae B728a chromosome, complete genome (HacA)

>NC_007005.1:5764268-5766655 Pseudomonas syringae pv. syringae B728a chromosome, complete genome (HacB)
